# Readability and Citation Sources of ChatGPT-Generated Responses to Lymphoma-Related Health Questions: Cross-Sectional Study

**DOI:** 10.2196/95067

**Published:** 2026-10-23

**Authors:** Corey H Basch, Tailisha González, Grace C Hillyer, Erin T Jacques, Charles E Basch

**Affiliations:** 1 William Paterson University Wayne, NJ United States; 2 Lehman College Bronx, NY United States; 3 York College, The City University of New York Jamaica, NY United States; 4 Teachers College, Columbia University New York, NY United States

**Keywords:** lymphoma, large language models, artificial intelligence, AI, online health information, health information–seeking behaviors, plain and clear language

## Abstract

**Background:**

Lymphoma is one of the most prevalent groups of cancers among people aged 15 to 29 years. Increasingly, patients with lymphoma and their caregivers use generative AI systems, supported by large language models (LLMs), to seek cancer-related online health information. Despite the need for plain and clear health information for clinical decision-making, there is a dearth of research on the readability and citation source classification of LLM-supported online health information.

**Objective:**

The objective of this study was to assess the reading level and reading ease of lymphoma-related health information using OpenAI’s GPT-5.2 model, as well as the citation sourcing of LLM-generated results.

**Methods:**

Questions were submitted to the LLM through an automated pipeline. The application code was written with Claude and run and hosted on Vercel. Standardized instructions were submitted to the LLM, asking for evidence-based sources with URLs to enable citation analysis. The final dataset included 392 citations garnered from 96 standardized lymphoma-related questions. Readability was assessed using the Flesch-Kincaid Grade Level and Flesch Reading Ease scores. Citation sources were classified into 9 predefined organization types, and topics included Hodgkin and non-Hodgkin lymphoma and clinical trials. Differences in readability were tested using linear models fitted to all 392 citations, with standard errors clustered by question. Estimated marginal means (EMMs) were used to summarize model-adjusted readability scores by citation source and topic. When the omnibus test was statistically significant, pairwise comparisons were performed using the Tukey-Kramer adjustment for multiple comparisons.

**Results:**

The Flesch-Kincaid reading levels of the ChatGPT responses by citation source and topic type were determined to be at the college or university level, well above the recommended sixth-grade level. Grade level did not differ by citation source (*P*=.58). The reading grade level of responses for Hodgkin lymphoma responses was statistically higher than that of clinical trial responses (mean 16.2, SD 3.4 vs 13.6, SD 2.5; *P*=.005*)*. Flesch Reading Ease scores across source and topic were found to be very difficult at the university graduate academic level and did not differ by source (*P*=.27) or topic (*P*=.17).

**Conclusions:**

Although LLMs frequently produce responses that include cited information, the readability of these outputs may limit accessibility for many patients and caregivers. These findings highlight the need for improved readability standards in AI-generated health information and reinforce that LLM outputs should complement, not replace, professional medical guidance.

## Introduction

Large language models (LLMs) are a type of generative AI that is becoming more prevalent in online health information (OHI)–seeking behaviors. Data from a national survey indicate that most OHI seekers considered LLM-supported platforms as useful as the information provided by their physicians [1]. Users often use AI tools alongside online searches, noting that they appreciate the speed and clarity, but remain cautious about accuracy and potential bias [2]. This pattern is echoed in national polling: 79% of US adults report that they are likely to search for online health answers, and nearly two-thirds consider AI-generated health information somewhat or very reliable, while attitudes remain mixed, with optimism about convenience tempered by concerns about the veracity of information [3,4]. Despite this growing reliance on LLMs for OHI, research on the quality, accessibility, and effectiveness of the content produced is limited, with one systematic review of AI in health literacy unable to determine the effectiveness of AI-generated OHI [5]. This gap in knowledge is important because low health literacy is a persistent barrier to effective health communication and many US adults’ reading comprehension skills remain below the level required to understand most medical materials [6,7]. This can complicate treatment, diagnosis, and participation in clinical trials [7,8].

Readability and source transparency are two factors known to shape how the public interprets health information [7]. Emerging evidence from other cancer contexts, such as recent findings on LLM responses to breast cancer questions, has raised concerns about missing evidence and potential ethical risks in AI-generated clinical content [9]. This issue is particularly salient when considering lymphoma, a group of blood cancers that includes both Hodgkin and non-Hodgkin subtypes, which together represent one of the most prevalent cancer groups among people aged 15 to 29 years [10]. The age group frequently seeks LLM-generated OHI [1] and reports the highest unmet information needs among patients with cancer [11]. Nationally, approximately 1 in 4 US adults who use AI tools for monthly health information and advice are less than 30 years of age, highlighting the potential role that LLMs could play in this population’s care [12]. Patients with lymphoma and their caregivers need timely, personalized information about diagnosis, treatment, and support, as this form of patient-centered cancer care has been linked to positive clinical outcomes [13]. Prior research has found that patients with blood cancer and their caregivers commonly seek information about referrals, financial assistance, second opinions, insurance coverage, diagnosis and prognosis, clinical trials, and social support [13]. The study examined individualized assistance provided by information specialists rather than the quality or accuracy of information generated by LLM-based platforms [13]. Because lymphoma affects age groups that commonly use AI tools and because these patients often have high information needs, lymphoma is a condition that merits greater attention.

To address this gap, the present study created questions focused on 3 clinical topics relevant to patients with lymphoma: Hodgkin lymphoma, non-Hodgkin lymphoma, and clinical trial participation. These topics reflect concerns that patients and caregivers have raised in past research [13]. The purpose of the current study was to assess the readability and citation source characteristics of ChatGPT-generated responses, powered by GPT-5.2 (OpenAI), to a standardized set of patient-directed lymphoma-related questions. This study intentionally evaluated the default, general-purpose configuration of GPT-5.2, without domain-specific fine-tuning or retrieval-augmented customization, as this reflects the tool as it is actually accessed and used by patients with lymphoma and their caregivers in real-world OHI-seeking behavior, rather than an idealized or clinically optimized deployment. By focusing on characteristics directly detectable to end users, this study aimed to provide an assessment of what patients encounter when using widely available AI tools to seek health information.

## Methods

### Overview

The principal objectives of this study were to assess readability and health information sourcing rather than the medical accuracy of the AI-generated responses. The methods used in this study build on prior research [14]. The 100 questions were developed a priori from patient and caregiver information needs identified in the literature; they covered Hodgkin lymphoma (30 questions), non-Hodgkin lymphoma (30 questions), and clinical trials (40 questions) to capture a broad range of topics related to diagnosis, treatment, prognosis, and trial participation. A total of 100 questions was selected to provide broad coverage while maintaining a feasible standardized sample. Data were collected on February 15, 2026.

### Data Extraction

#### Phase 1

The data extraction involved 2 phases. The first phase began by storing all 100 questions on a backend cloud database [15,16] and submitting them to ChatGPT through an automated pipeline. The Node.js (OpenJS Foundation) [17] application code for that pipeline was written with Claude (Anthropic PBC) [18] and run and hosted on Vercel (Vercel Inc) [19]. Each prompt included a standardized instruction asking the LLM to provide evidence-based sources with URLs, enabling downstream citation analysis. All responses were generated using OpenAI’s GPT-5.2 model [20], which was selected because it is the default model available to ChatGPT users, including those on the free tier. Each question was submitted as a separate, independent prompt so that responses were generated without influence from previous questions or conversation history.

The temperature parameter in the application programming interface was set to 0 so that there would be consistency in ChatGPT’s generated responses, ensuring reproducibility of the study. The pipeline saved the full response along with any cited URLs. Each cited URL was stored as a separate record linked to its originating response, enabling analysis at both the response and citation levels. URLs were removed from the response text before analyzing readability because long web addresses can artificially make sentences and words look more complex than they are in actuality.

Readability was assessed using the Flesch-Kincaid Grade Level (FKGL) and Flesch Reading Ease (FRE) [21] formulas applied to cleaned response text. These scales have been used previously to examine patient comprehension of health information [22]. FKGL estimates the US school-grade reading level required to understand the text, whereas FRE scores typically range from 0 to 100, with higher values indicating easier readability. FKGL scores were compared against the American Medical Association’s recommendations that patient-facing materials be written at or below a sixth grade reading level [23]. The script used to calculate FKGL and FRE scores from response text was developed with Claude [18].

#### Phase 2

In the second phase, each cited URL was classified into 1 of 9 predefined organization types: government agencies, academic or research institutions, peer reviewed journals, professional associations, commercial or industry entities, news or media organizations, hospitals or health systems, encyclopedias, and other or unspecified health information sources. These categories distinguished sources based on the type of organization responsible for producing the content.

To facilitate the analysis of each URL’s organization name and type, a Google Apps Script was written with Claude [18] and subsequently run in Google Sheets. Classification proceeded through 3 stages: matching domains against a curated database of approximately 80 known health organizations, evaluating top-level domains (eg, .gov and .edu) to infer institutional type, and reviewing web page content for unmatched sources using publisher and metadata information. Sources that did not clearly fit a standard category were labeled “Other or Unspecified Health Information Sources.” Each citation was classified independently.

Prior to analysis, all extracted URLs and source classifications were manually reviewed for accuracy. Additional human validation procedures included verifying active links, contextually relevant web pages, and correct classifications. Citations were excluded if they were nonfunctional, inaccessible, unrelated to health topics, or represented clearly hallucinated AI-generated references. Responses were excluded only if all cited URLs were invalid or inaccessible, regardless of the quality of the information provided. The full pipeline source code is publicly available at GitHub (Microsoft Corp) [24].

### Data Analysis

We examined whether response readability differed according to the type of source cited in the response and across topics. Each AI-generated response was evaluated for readability using the FKGL and FRE measures. Because readability reflects characteristics of the complete response text rather than individual citations, each response-level FKGL and FRE score was linked to each retained citation within that response. This produced a citation-level analytic dataset (N=392), in which citations originating from the same response shared the same readability score. The dataset was used to examine differences in readability according to citation source type and topic category.

Because citations from the same response shared 1 readability score, observations were not statistically independent. We therefore compared readability using linear models fitted to all 392 citations, with standard errors clustered by question (96 clusters). Separate models were fitted for FKGL and FRE. Citation source type was modeled as a categorical predictor adjusted for topic, and topic was modeled as a categorical predictor. A random-intercept linear mixed-effects model was considered but not used because readability had no within-question variation (intraclass correlation=1), which made that model singular. Omnibus effects were reported as *F* tests with *df*.

FKGL scores exhibited a statistically significant departure from normality (Shapiro-Wilk *P*<.001), although the distribution showed only modest positive skewness (skewness=0.91). The skewness was largely attributable to 1 extreme response; after exclusion of this observation, skewness was 0.06. Therefore, both means (SD) and medians (IQR) were reported for FKGL.

Because the linear models were relatively robust to moderate departures from normality, inference was based on these models. Sensitivity analyses excluding the extreme observation and using a Kruskal-Wallis test on response-level readability scores produced the same substantive conclusions.

Estimated marginal means (EMMs) with 95% CIs were derived from the fitted models. Pairwise comparisons were made from the EMMs using Tukey-Kramer adjustment for multiple comparisons when the omnibus test was statistically significant. We reported the full pairwise results for the only analysis with a significant omnibus test. Statistical significance was defined as *P*<.05. Analyses were conducted using SPSS (version 30.0; IBM Corp) [25].

### Ethical Considerations

The institutional review board at William Paterson University determined that this study constituted non–human subjects research and granted a waiver (IRB-FY2026-135). Informed consent did not apply to the study.

## Results

A total of 100 lymphoma related questions were submitted to GPT-5.2, generating 100 independent responses. The raw dataset expanded these responses into citation-level records, resulting in 548 citation sources. After validation and removal of 156 (28.5%) incorrect or inaccessible links, 392 (72%) verified citations remained in the cleaned analytic dataset.

Exclusion of the broken or inappropriate links resulted in the removal of 2 Hodgkin and 2 non-Hodgkin lymphoma questions because all associated citations for those responses were invalid or inaccessible. The final dataset included 392 citations garnered from 96 questions (Table 1). Distribution of the citations roughly aligned with the proportion of questions asked about each topic (eg, 40/96, 41.7%, of questions related to clinical trials, and 173/392, 44.1%, of the citations were accrued from this topic). Government citations far exceeded information from other sources, with 53.6% (210/392) citations overall, and by discussion topic, whereas only 5.4% (21/392) of overall citations were from encyclopedias. Citations from peer reviewed journals and professional associations were common for Hodgkin and non-Hodgkin lymphoma, with Hodgkin lymphoma responses more often citing encyclopedias, but responses for clinical trials were predominantly government and other or unspecified sources rather than peer reviewed journals or professional associations.

Readability scores were calculated from the text of the ChatGPT responses generated for each question after removing embedded URLs. The mean and median FKGL scores of the ChatGPT responses (Table 2) were at the college or university level overall but did not differ significantly (*P*=.58). No pairwise comparisons were statistically significant (*P*≥.76).

By topic, FKGL scores varied significantly (*P*=.007), and all mean and median scores were at the college or university level (Table 3). Pairwise comparisons (Table 4) indicated significant grade level readability differences between Hodgkin and clinical responses (*P*=.005); however, both scores were at the college or university level.

**Table 1:** Citations (N=392) by source in ChatGPT-generated responses to questions about Hodgkin lymphoma (n=28), non-Hodgkin lymphoma (n=28), and clinical trials (n=40)^a^.

	Citation source, n (%)					
	Total^b^	Government^c^	Peer reviewed journal^c^	Professional association^c^	Encyclopedia^c^	Other or unspecified^c^
Hodgkin lymphoma	113 (28.8)	43 (38.1)	28 (24.8)	25 (22.1)	12 (10.6)	5 (4.4)
Non-Hodgkin lymphoma	106 (27)	62 (58.5)	19 (17.9)	25 (23.6)	0 (0)	0 (0)
Clinical trials	173 (44.1)	105 (60.7)	16 (9.2)	16 (9.2)	9 (5.2)	27 (15.6)
Total	392	210 (53.6)	63 (16.1)	66 (16.8)	21 (5.4)	32 (8.2)

^a^Of 9 predefined citation source types, only 5 appeared in the dataset.

^b^Based on column total.

^c^Based on row total.

**Table 2:** Flesch-Kincaid Reading Level scores^a^ by citation source (N=392 citations)^b^.

	Citation source^c^					
	Overall	Government	Peer reviewed journal	Professional association	Encyclopedia	Other or unspecified
n	392	210	63	66	21	32
Mean (SD)	14.7 (3.0)	14.4 (2.8)	15.4 (3.3)	14.8 (2.8)	16.7 (4.5)	13.7 (1.7)
Median (IQR)	14.4 (12.8-16.5)	14.2 (12.5-16.6)	15.2 (14.1-16.2)	15.3 (13.2-16.6)	15.8 (12.9-21.4)	13.3 (12.2-15.7)
Range	8.3-29.7	8.3-29.7	8.3-17.4	8.3-20	8.3-21.4	11.3-17.9
EMM^d^ (95% CI)		14.5 (13.8-15.3)	15 (14-16.1)	14.5 (13.6-15.4)	16.3 (13-19.7)	14.4 (13.6-15.1)

^a^0 to 6: elementary school (basic reading); 7 to 9: middle school to early high school (general audience or plain English); 10 to 12: high school level; and 13+: college or university level.

^b^Of 9 predefined citation source types, only 5 appeared in the dataset.

^c^*F*_4,95_=0.73; *P*=.58; Tukey-Kramer *P*≥.76.

^d^EMM: estimated marginal mean.

**Table 3:** Flesch-Kincaid Reading Level scores^a^ by topic category (N=392 citations).

	Topic category^b^		
	Hodgkin lymphoma	Non-Hodgkin lymphoma	Clinical trials
			
n	113	106	173
Mean (SD)	16.2 (3.4)	14.8 (2.6)	13.6 (2.5)
Median (IQR)	15.8 (15.1-16.7)	14.8 (13.2-16.9)	13.6 (12.1-15.6)
Range	9.9-29.7	9.9-21	8.3-17.9
EMM^c^ (95% CI)	16.2 (14.9-17.5)	14.8 (13.7-15.9)	13.6 (12.7-14.6)

^a^0 to 6: elementary school (basic reading); 7 to 9: middle school to early high school (general audience or plain English); 10 to 12: high school level; and 13+: college or university level.

^b^*F*_2,95_=5.25; *P*=.007.

^c^EMM: estimated marginal mean.

**Table 4:** Pairwise comparisons.

Pair	Difference (95% CI)	Tukey-Kramer *P* value
Hodgkin and non-Hodgkin	1.40 (–0.65 to 3.45)	.24
Hodgkin and clinical trials	2.56 (0.66 to 4.46)	.005
Non-Hodgkin and clinical trials	1.16 (–0.59 to 2.91)	.26

The overall mean FRE score of ChatGPT responses and means by citation source were interpreted as “very difficult, university graduate or academic level” (Table 5) but were not significantly different by citation source. Responses associated with encyclopedia sources had the numerically lowest mean score, but differences were not significant. No pairwise comparisons were statistically different. FRE scores also did not vary by topic (*P*=.17), with no significant differences observed in pairwise comparisons (*P*≥.14) (Table 6).

**Table 5:** Flesch Reading Ease scores^a^ by citation source (N=392 citations)^b^.

	Citation source^c^					
	Overall	Government	Peer reviewed journal	Professional association	Encyclopedia	Other or unspecified
n	392	210	63	66	21	32
Mean (SD)	22.7 (12)	24.1 (12.5)	19.2(9.2)	23.3 (11.7)	17.5(15.7)	22.7 (8.5)
Range	1.5-50.3	1.5-50.3	1.5-49	3.3-49	2.2-49	5.6-38.1
EMM^d^ (95% CI)		23.7 (20.3-27.1)	20.1 (16.9-23.4)	24 (20.2-27.8)	18.3 (6.2-30.4)	21.3 (17-25.6)

^a^90 to 100: very easy (fourth-grade level); 80 to 90: easy (fifth-grade level); 70 to 80: fairly easy (sixth-grade level); 60 to 70: standard (seventh- to eighth-grade level; ideal for most web content); 50 to 60: fairly difficult (high school level); 30 to 50: difficult (college level); and 0 to 30: very difficult (university graduate or academic level).

^b^Of 9 predefined citation source types, only 5 appeared in the dataset.

^c^*F*_4,95_=1.32; *P*=.27; Tukey-Kramer *P*≥.29.

^d^EMM: estimated marginal mean.

**Table 6:** Flesch Reading Ease scores^a^ by topic category (N=392 citations).

	Topic category^b^		
	Hodgkin lymphoma	Non-Hodgkin lymphoma	Clinical trials
n	113	106	173
Mean (SD)	19.2 (11.2)	22.7 (12.2)	25 (11.8)
Range	2.2-45.3	1.5-45	6.2-50.3
EMM^c^ (95% CI)	19.2 (14.8, 23.5)	22.7 (17.1, 28.3)	25 (20.8, 29.2)

^a^90 to 100: very easy (fourth-grade level); 80 to 90: easy (fifth-grade level); 70 to 80: fairly easy (sixth-grade level); 60 to 70: standard (seventh- to eighth-grade level; ideal for most web content); 50 to 60: fairly difficult (high school level); 30 to 50: difficult (college level); and 0 to 30: very difficult (university graduate or academic level).

^b^*F*_2,95_=1.82; *P*=.17; Tukey-Kramer *P*≥.14 (all adjusted *P* values).

^c^EMM: estimated marginal mean.

## Discussion

### Principal Findings

This study examined the readability and reading ease of LLM-supported OHI and found that ChatGPT responses consistently exceeded national averages for adult literacy. Readability did not differ significantly by citation source type: responses citing government, peer reviewed, professional association, encyclopedic, and other sources were all written at a college level or above. Readability did differ by topic, with responses to Hodgkin lymphoma questions written at a higher grade level than responses to clinical trial questions, although reading ease did not differ significantly by topic. The FRE score for “very difficult” is 0 to 30. The recommended score for adult health literacy is 80+. Despite the Plain Writing Act of 2010 requiring federal executive branch agencies to write clear, concise, and well-organized communication that can be read and understood easily, this study on ChatGPT responses highlights that difficult reading levels were present regardless of the type of source cited. There is an urgent need for LLM architects to consider readability and reading ease due to the growing concentration of users tasking LLMs to synthesize complex health information. Citation sourcing also varied by topic: government sources predominated overall and especially for clinical trial questions, whereas responses to Hodgkin and non-Hodgkin lymphoma questions more often cited peer reviewed journals and professional associations.

### Comparison With Prior Work

Our findings support previous research highlighting flaws in LLM use that may contribute to the dissemination of misinformation, such as broken links, fabricated references, and inaccurate components [26]. The findings of this study suggest that while LLMs frequently provide cited information, the accessibility of that information remains limited. Although most responses in our sample included references, predominantly from government and institutional sources, the readability of the generated content often substantially exceeded recommended sixth grade reading levels for patient education materials. This lack of alignment with established plain language standards is concerning, as health information that is not written in clear, accessible terms may undermine comprehension, particularly among individuals with limited health literacy.

Research indicates that, although LLMs may enhance efficiency and personalization in health contexts, their integration requires careful oversight given documented risks of bias, misinformation, transparency, and legal uncertainty [27]. This study supports these recommendations for generative AI development. A separate study examined the specificity and currentness of ChatGPT’s responses to questions about hematologic malignancies with input from oncologists and found that the LLM was unable to provide specific information for patient inquiries and emerging therapies, largely due to AI’s reliance on datasets that may become obsolete in an ever-changing clinical landscape [28]. Beyond issues of accuracy, evidence from natural language processing research demonstrates that linguistic indicators of limited health literacy are associated with older age and poorer clinical outcomes, suggesting that LLMs that do not consistently adhere to plain language principles may risk exacerbating age- and literacy-related disparities in health outcomes [29].

Interestingly, recent research by Will et al [30] demonstrated that LLMs, such as Claude, Gemini, and ChatGPT, may also serve as tools to improve the accessibility of patient materials. In their cross-sectional analysis, the authors found that LLMs were able to substantially reduce complexity and shorten text length while preserving accuracy and understanding. Sporadic inaccuracies were observed, which reinforces our recommendation for careful human review of LLM-generated medical information [30].

A recent scoping review of 201 studies found that LLMs are increasingly being used for OHI, with applications spanning the creation of educational content, explanation of medical information and instructions, the delivery of personalized treatment and lifestyle guidance, and support for enhanced communication [31]. Despite these advances, substantial concerns persist regarding the accuracy, readability, potential bias, transparency, and ethical implications of LLM generated content [31]. The current study supports increased regulation of generative AI development and safeguards for the public. Generative AI’s predilection for producing content that resembles high-quality, polished, well-structured output that is perceived as more valid or accurate than human-generated information may lead patients to make misinformed health care decisions. Several researchers have already warned against the dangers of this AI related pitfall [32].

### Implications

This study provides valuable insight for future research on LLMs for OHI. For LLMs to serve as tools to close gaps in health literacy, additional training is required to facilitate effective multistep health information–seeking behavior. Studies have demonstrated that ChatGPT may be a reliable tool for explaining complex medical terms in certain contexts, such as following a diagnostic appointment with a health care provider. In a study comparing medical practitioners’ responses for explaining complex medical terminology to ChatGPT’s explanations, the LLM-supported platform demonstrated superior reliability in translating medical terms into understandable language, exceeding the capabilities of the most experienced physicians in the study [33]. In this example, LLMs served as auxiliary tools, rather than diagnostic alternatives, providing plain language explanations that may be used as part of clinical decision making in consultation with a health care professional. These approaches to OHI-seeking behaviors may also protect patients from AI-generated hallucination, fabricated references, and misleading citations [34]. Future research is required that examines these multilayered contexts for LLM-supported OHI.

### Limitations

Several limitations should be noted. This study only checked how the AI responses were written (2 specific features), not whether the medical information in them was correct or complete. Future research should incorporate a focused review or sensitivity analysis of a subset of responses with input from oncologists, patients, caregivers, and other content experts to evaluate hallucination, fabrication, or clinically meaningful inaccuracies, as these dimensions of response quality are not captured by readability or citation sourcing alone. The questions were developed by a research team rather than members of the public, which may introduce bias in terms of framing, priorities, and underlying assumptions that could influence responses generated by the LLM. In addition, all responses were generated by a single model; therefore, findings cannot be generalized to other LLMs or to real world interactions that may involve follow-up questions or personalized dialogue. This study also evaluated only the default, general purpose configuration of GPT-5.2; future research should test whether AI systems trained or adapted for medical topics can produce lymphoma information that is both accurate and easier to read. Readability was calculated once per response and applied to every citation from that response, so scores are not independent across citations. Although we accounted for this by clustering on question, source-type comparisons rely on variation between responses, and power was limited for smaller groups, such as encyclopedia sources (n=21). The classification of citation sources was automated and, although validated through manual review, may not fully capture more nuanced distinctions in source relevance or informational quality. Finally, the exclusion of broken or inaccessible links, while necessary for a cleaner analysis, suggests that the true proportion of unreliable citations in unfiltered LLM outputs may be higher than reported here. Nevertheless, to our knowledge, this study is the first to examine the readability of LLM-generated responses for patients and caregivers seeking lymphoma-related health information.

### Conclusions

OHI seeking behaviors will continue to be shaped and molded by LLMs over the coming decade. This burgeoning field of research should focus on integrating LLMs in health literacy interventions so that patients are able to glean the full benefits of this technology and maximize its capacity to break down complex health terminology as part of their information seeking and decision-making. Health education campaigns must simultaneously stress that LLMs are not a substitute for medical expertise from a health care professional and that these programs are prone to errors and the production of homogenized outputs, hallucination, fabrication, and incomplete information.

## Abbreviations

EMM: estimated marginal mean
FKGL: Flesch-Kincaid Grade Level
FRE: Flesch Reading Ease
LLM: large language model
OHI: online health information

## Data Availability

The code is available on GitHub [24], and the datasets generated and analyzed are available upon reasonable request.
